# Mapping Scientific Knowledge on Body Composition and Anthropometry in Soccer: A Bibliometric Analysis of the Last 35 Years (1990–2025)

**DOI:** 10.3390/sports14080349

**Published:** 2026-08-12

**Authors:** Juan David Paucar-Uribe, Boryi A. Becerra-Patiño, Aura D. Montenegro-Bonilla, Armando Monterrosa-Quintero, Jorge Olivares-Arancibia, Daniel Rojas-Valverde, Rodrigo Yáñez-Sepúlveda, José Francisco López-Gil

**Affiliations:** 1 Faculty of Physical Education, National Pedagogical University, Bogota 480100, Colombia; jdpaucaru@upn.edu.co (J.D.P.-U.); babecerrap@pedagogica.edu.co (B.A.B.-P.); admontenegrob@upn.edu.co (A.D.M.-B.); 2 Altius Performance Laboratory, Physical Education and Sports Program, Universidad Surcolombiana, Neiva 410001, Colombia; adomonterrosa@gmail.com; 3 AFySE Group, Research in Physical Activity and School Health, School of Physical Education, Faculty of Education, Universidad de las Américas, Santiago 7500975, Chile; jorge.olivares.ar@gmail.com; 4 Center for Research, Development and Innovation in Health and Sport (CIDISAD), School of Human Movement Sciences and Quality of Life (CIEMHCAVI), National University, Heredia 863000, Costa Rica; drojasv@hotmail.com; 5 Faculty of Education and Humanities, School of Sport Sciences, Universidad Andres Bello, Viña del Mar 2520000, Chile; rodrigo.yanez.s@unab.cl; 6 Department of Sport Sciences, Faculty of Sport and Health Sciences, Fit Generation Research Institute, AD500 Andorra la Vella, Andorra; 7 School of Medicine, Universidad Espíritu Santo, Samborondón 092301, Ecuador; 8 Vicerrectoría de Investigación y Postgrado, Universidad de Los Lagos, Osorno 5290000, Chile

**Keywords:** anthropometric factors, measurement methods, sports sciences, sports performance, ISAK, body composition

## Abstract

**Background:** Anthropometric analysis has become a key tool for analyzing body composition to assess, monitor, and track structural changes in soccer players. **Objective:** The objective of this study was to analyze the scientific literature on body composition and anthropometry in soccer using a bibliometric analysis. **Material and Methods:** The following databases were consulted: Scopus, Web of Science (WoS), PubMed (Medline), SportDiscus, and the ScienceDirect publishing platform. The study included documents published between 1990 and 2025. Data was selected following the Preferred Reporting Items for Systematic Reviews and Meta-Analyses (PRISMA) guidelines statement flowchart and subsequently analyzed using Bibliometrix and R software to identify keywords, frequency of occurrence, journal analysis, countries, and other bibliometric indicators. **Results:** A total of 384 documents met the inclusion criteria. Spain, Italy, Brazil and Portugal stand out as the countries with the highest output. These countries are associated with the most representative authors, led by Suarez-Arrones, L; Nikolaidis, PT; and Campa, F., who are in turn associated with the most representative concepts: “Male”, “Adolescent” and “Athletic performance”. The most prevalent concepts are population-based: “Adolescent” and “Young adult”. The top three organizations in terms of contributing to the scientific study of body composition and anthropometry were the University of the Basque Country, Pablo de Olavide University, and the University of Coimbra. It can be observed that most of the literature has focused on youth (29.16% of the total documents), followed by elite players (22.91%) and professionals (14.84%), where the focus is on those aged 14 to 19 years and playing at highly competitive levels, seeking peak performance. **Conclusions:** Methodological heterogeneity in evaluation protocols constitutes the main structural shortcoming in this field: 32.29% of studies do not specify their protocols, which compromises the comparability of the results. The widespread adoption of international standards, such as the International Society for the Advancement of Kinanthropometry (ISAK) methodology, along with a requirement to indicate the instrument used and the evaluator’s certification level, is a priority for improving the quality of scientific communication in future research.

## 1. Introduction

Structural changes in the human body have been a subject of interest throughout history [1]. The first records of body measurements, dimensions, and proportions were compiled as early as 1920 [2]. Different measurement methods have been used to monitor these variables [3]. One of the most widely used approaches in clinical practice is bio-impedance spectroscopy [4]. Other approaches, such as dual-energy X-Ray absorptiometry (DXA) [5,6] and anthropometry [7], have existed for more than 30 years but have limited use. However, some of these instruments have limitations in sports science. Anthropometry is one of the most widely used tools due to its low cost, ease of use, and utility based on scientific estimates [7]. As a result, it has become a useful tool, as it enables the transfer of data to the athletes’ actual playing field under real game conditions, which requires the implementation of standardized protocols such as those developed by certain scientific societies for the collection of reliable data and the determination of physical characteristics through various indices and data, such as somatotypes associated with human body profiles and the monitoring of athletes.

Furthermore, given the wide range of body composition analysis techniques [6] and, to the best of our knowledge, the lack of any previous compilation of a substantial number of studies, we analyzed the variety of available methods to identify which ones are most commonly used and the proposed methodological combinations for assessing body composition in soccer. The latter approach (anthropometry) is responsible for conducting assessments using specialized metrics, such as skinfold thickness, body girth, and bone breadth, to gather individual anthropometric traits, including length, height, and basic measurements (weight, stature, arm span, and sitting height) [8,9], yielding information on body composition variables (lean mass, bone mass, fat mass, and residual mass) [10], somatotypes, and phantom strategies (Z-score) [11,12,13], among many others. Furthermore, the estimation of equations is fundamental for the indirect estimation of physical variables [14,15] and, in the same way, waist-to-height ratio, waist circumference, body mass index (BMI) [16], and other metrics associated with sports performance.

Therefore, anthropometry and kinanthropometry have become reliable scientific tools for the assessment, monitoring, and ongoing analysis of physical and compositional changes as well as specific athletic goals [17]. It is very important that some studies have focused on the training and career prospects of professionals who perform these measurements in the sports field [18], with the International Society for the Advancement of Kinanthropometry (ISAK) being responsible for implementing global standardization [8]. In a sport like soccer, athletic performance depends on multiple variables [19], with physical characteristics allowing the creation of athlete profiles that are fundamental for athlete selection [20], and anthropometry is a key tool that has allowed monitoring of the physical performance of soccer players [21], as well as the impact that diets have during the competition season [22], and establishment of comparisons with different physical capacities associated with physical performance [23].

Technological advances have led to an increase in scientific production and available information [24], and, currently, there is a wide variety of topics related to research on soccer and anthropometry [25,26,27], generating a broad literary landscape that has yet to be fully explored. Among the main topics are related studies, such as those by Petri et al. [9], who compared ISAK standards between sexes and found that male athletes have a mesomorph–ectomorph profile, while women’s somatotypes are classified as balanced mesomorphs. However, it is important to note that female samples are subject to greater difficulties due to the menstrual cycle [28], leading to further articles discussing these results [29].

On the other hand, there is currently interest in identifying differences across playing positions in physical, physiological, and technical variables [30] and anthropometric variables [31], recognizing that each position presents distinct characteristics [32]. The results show that outfield players (defenders, midfielders, and forwards) have lower body fat percentages than goalkeepers; in fact, these results also vary according to each player’s competitive level [31,33]. This has led to articles with diverse characteristics, such as the study by Becerra-Patiño et al. [34], which considered many variables that anthropometry can provide, allowing the application of factor analysis and multivariate methods associated with performance and body composition indicators in the same study. This wide range of studies highlights the need to understand scientific findings on anthropometry as applied to soccer. Unlike in other literature reviews, bibliometric analysis allows us to map different variables in the existing literature, focusing not on methodological quality but on the most significant scientific advances, countries, authors, and affiliations; to identify thematic trends; and to highlight, within the vast body of the scientific literature, knowledge gaps that could lead to new research opportunities. The objective of this study was to conduct a bibliometric analysis to analyze scientific output in the field of anthropometry and body composition in soccer between 1990 and 2025 using the Bibliometrix R tool (Bibliometrix Package: Version 5.4.1, Graphical Interface: Biblioshiny).

## 2. Materials and Methods

### 2.1. Study Design

This study uses bibliometric analysis as a research method for analyzing scientific output [35] and the Preferred Reporting Items for Systematic reviews and Meta-Analyses (PRISMA) guidelines. Following the classification established by Powell et al. [36], it is a theoretical and retrospective study that aims to systematize, group, and analyze many documents on a specific topic.

### 2.2. Sources of Information

The search strategies consider the following characteristics:

Date: 1 September 2025

The search was conducted on the following databases and scientific publication platforms: Scopus, Web of Science (WoS), PubMed (Medline), ScienceDirect and SportDiscus. We aimed to retrieve as many articles as possible on multidisciplinary topics in sports science related to the research topic, with broad international coverage (Scopus and WoS), based on the biomedical literature (PubMed) and relevant sports science precedents (SportDiscus), drawing on information from the most important publishers (ScienceDirect). The documents ranged from the first report recovered in 1990 to 2025.

### 2.3. Data Search and Extraction Strategy

Due to the lack of search equations for bibliometric analysis, the PICOS strategy was chosen (participants, intervention, comparison, outcomes and study design) for data collection, following the guidelines for preparing reviews [37]. However, after applying various search strategies, we modified the PICOS strategy to align it with the criteria used in other bibliometric studies [38,39]. Our approach is unlike traditional bibliometric analysis searches, which involve creating terms and Boolean operators without a logical order, a practice deliberately employed by researchers that introduces bias in the retrieval of documents stored in databases. In addition, a single search equation was used for all databases to ensure methodological consistency and to ensure that the results would be comparable in the screening of information.

The Boolean operators “AND” and “OR” were used to group the terms. A similar procedure was followed for each database. Before the construction of the final search phrase for each database, different combinations were tested with the following list of keywords: (“soccer” [All fields]), (“soccer players” [All fields]), (“women’s soccer” [All fields]), (“anthropometric” [All fields]), (“ISAK” [All fields]), (“anthropometry” [All fields]), (“body mass index” [All fields]), (“body composition” [All fields]), (“somatotype” [All fields]), (“morpho* characteristics” [All fields]), (“morphofunctional* variables” [All fields]). Finally, after testing various search queries in the databases, and with the aim of identifying the most relevant query in terms of consistency, without introducing bias based on these terms, we developed the following search strategy: (soccer OR “soccer players” OR “women’s soccer”) AND (ISAK OR anthropometric OR anthropometry OR “body mass index” OR “body composition” OR somatotype OR “morphological characteristics” OR “morphofunctional variables”). The search results were obtained by including all studies published up to 1 September 2025.

The search was conducted independently by two researchers (J.D.P.-U. and B.A.B.-P.) to determine the total number of documents included. The percentage of disagreement between the two researchers did not exceed 5% of all the studies included. Any disagreements were resolved by a third, independent reviewer who analyzed the documents to define the list of studies included. Initially, the reviewer downloaded the databases in Comma-Separated Values (CSV) and Excel formats to compile a final matrix. Duplicate documents were identified and removed based on the title, DOI, publication year, and authors; they were manually deleted using an Excel spreadsheet to ensure that each of the retrieved records was correctly removed. The following inclusion criteria were established: (i) studies published between 1990 and 2025; (ii) studies focused on soccer players or that included both sexes; (iii) for articles and reviews, those published in journals indexed in the Journal Citation Reports (JCR) or the Scimago Journal Rank (SJR) were considered; additionally, on an exceptional basis, book chapters, conference papers, or letters related to the study’s objective were accepted; (iv) studies with no restrictions regarding the type of published document or language; and (v) studies with no restrictions regarding the technique used to assess body composition. The exclusion criteria were (i) documents comparing soccer with another sport; (ii) studies dealing with indoor soccer; and (iii) articles dealing with soccer but focused on referees.

Ultimately, only classified studies were included. Furthermore, there was no discrimination based on year of publication, language, or study design. In total, we obtained five files—file 1 (Scopus, n: 2291), file 2 (Web of Science, n: 1885), file 3 (PubMed, n: 1693), file 4 (ScienceDirect, n: 2445), and file 5 (SportDiscus, n: 1986)—accounting for a total of 10,300 included documents. Although file 4 is not a database but rather a scientific publishing platform, its inclusion made it possible to retrieve relevant articles on the topic to identify additional documents and collect full-text documents. The five files were combined into a single text file (CSV) using Microsoft Excel with comma-delimited instructions, which allowed for the unification of the database and its subsequent processing using the Bibliometrix R tool.

**Figure 1:**
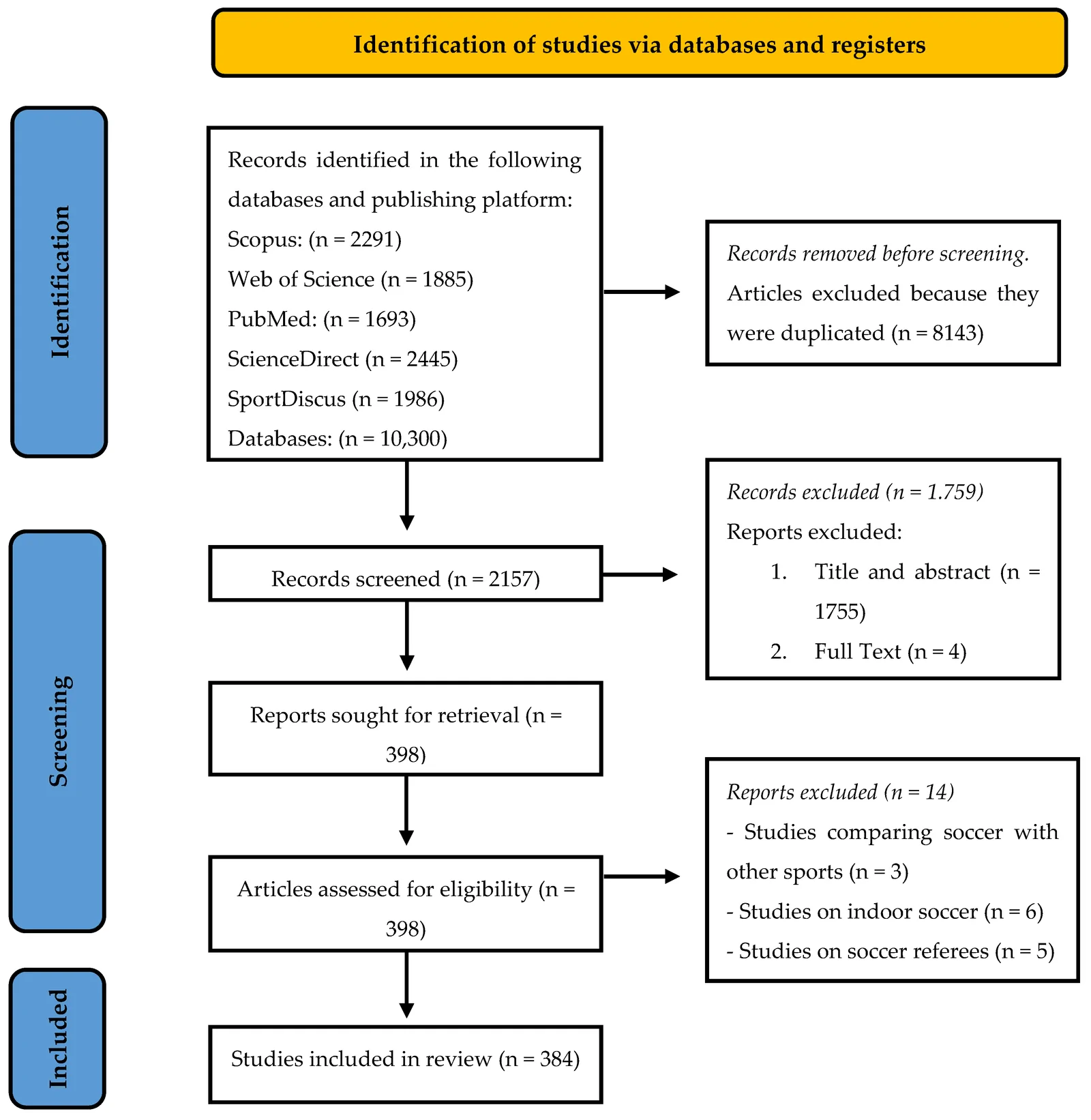
Flow diagram of bibliometric analysis.

Initially, 10,300 documents were identified and subjected to a metadata review process to eliminate duplicates, documents for which full access was not allowed, and studies not relevant to the study topic. When it was not possible to obtain the full-text articles through institutional subscriptions or open access, attempts were made to contact the authors directly or search for the documents on the ResearchGate platform, as has been done in other studies [10]. After reviewing the documents, we included 384 documents, following the eligibility criteria established for this study (Figure 1). Next, following the screening process, the included documents were analyzed through a review process using the analysis variables, such as affiliations, authors, titles, and keywords, so that the bibliometrix software (Bibliometrix Package: Version 5.4.1, Graphical Interface: Biblioshiny, Naples, Italy) product could properly interpret each document, avoiding duplicate bias through the standardization of the information.

### 2.4. Data Analysis

The R program Bibliometrix was used to systematize and visualize the data in R Statistical Software (Vienna, Austria, version 4.5.2), a programming language for statistical calculations and graphics. It is an integrated open-source tool that allows for a comprehensive analysis of trends in the scientific literature [40,41]. We analyzed three bibliometric dimensions: (i) scientific production and dissemination (which includes the number of articles published per year, journals, countries, citations, and authors); (ii) scientific collaboration (which includes national and international collaborative articles, as well as non-collaborative articles); and (iii) bibliometric evaluation at the individual level. Thus, the bibliometric analysis is based on the consideration of the following laws: (i) Price’s law to determine the growth in scientific output [42], in this case, the number of published documents and citations in relation to the years, as well as the total number of citations received; (ii) Lotka’s law to identify authors who have published more studies [43]; and (iii) Zipf’s law to establish the occurrence of the most frequently used keywords [44]. (iv) Bradford’s Law of Concentration of Science [45] was used to identify the journals most dedicated to the topic and those with the highest citation rates. This analysis will help us identify the most prolific and most cited journals in this field. Indicators of dimensionality, occurrences, word frequencies, co-authorship, and organizations were also analyzed within the network analysis to identify patterns of academic productivity.

## 3. Results

### 3.1. Evolution of the Number of Documents and Citations

**Figure 2:**
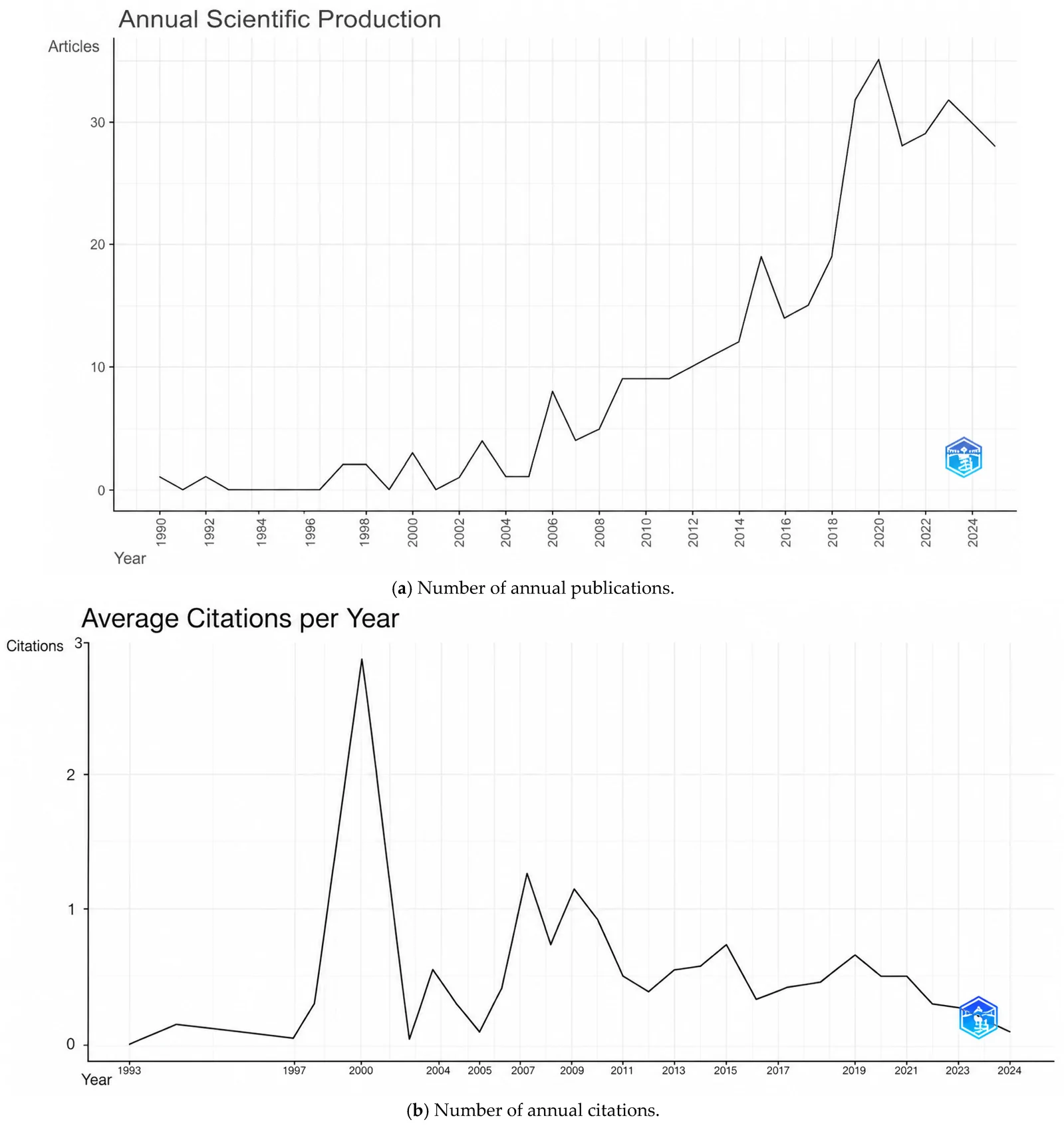
Evolution of the number of annual publications and citations.

Scientific output over time, measured by the number of documents published annually between 1990 and 2025, shows an initial peak in growth in 2006, followed by an increase in publications, with 2015 marking the second most significant increase; in 2018 and 2020, the highest scientific productivity was recorded, with exponential growth that aligns with Price’s law, reaching the peak of scientific output in this research area, and a stabilization of output can be observed between 2020 and 2025, marking the final phase of consolidation (Figure 2a). At the same time, a fluctuation was observed in the total number of citations, with the year 2000 registering the highest number of citations and low levels since 2015 (Figure 2b).

### 3.2. Types of Documents

There are various types of documents that enable the investigation of body composition and anthropometric characteristics in soccer. Most documents are articles, which represent almost all the research generated and retrieved (Table 1).

### 3.3. Thematic Trends

**Figure 3:**
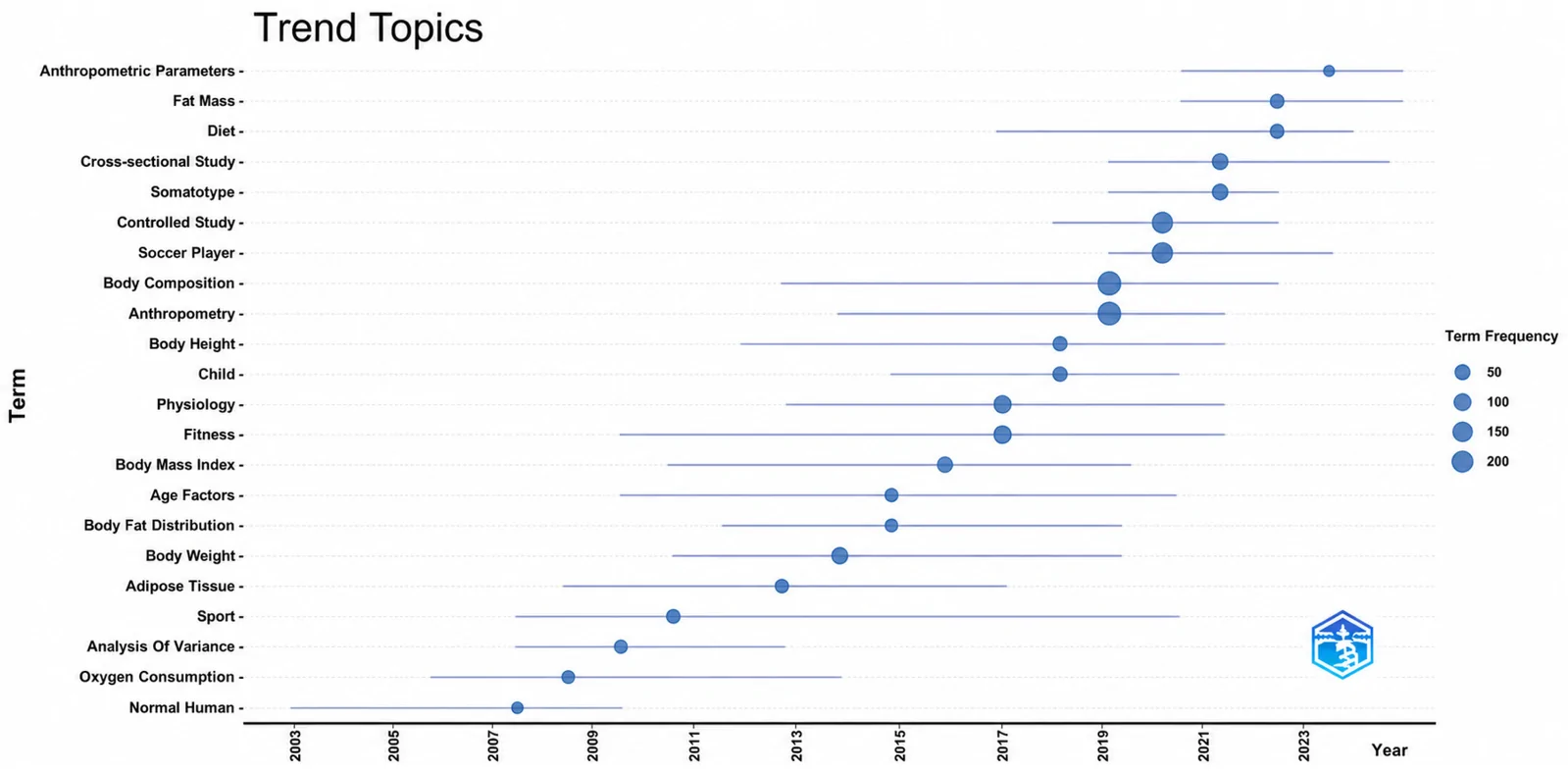
Trend themes. The X-axis represents the year, and the Y-axis represents cumulative occurrences in response to keywords.

The analysis of the most relevant topics detailed in Figure 3 shows that the most recent findings from studies on anthropometry and body composition in soccer players relate to nutritional factors such as diet, the sporting stage of youth players, and cross-sectional research designs. Furthermore, the most representative topics emerged between 2019 and 2020, with “body composition” and “anthropometry” being the most prominent in 2019, and “controlled study” and “soccer players” leading in 2020.

**Figure 4:**
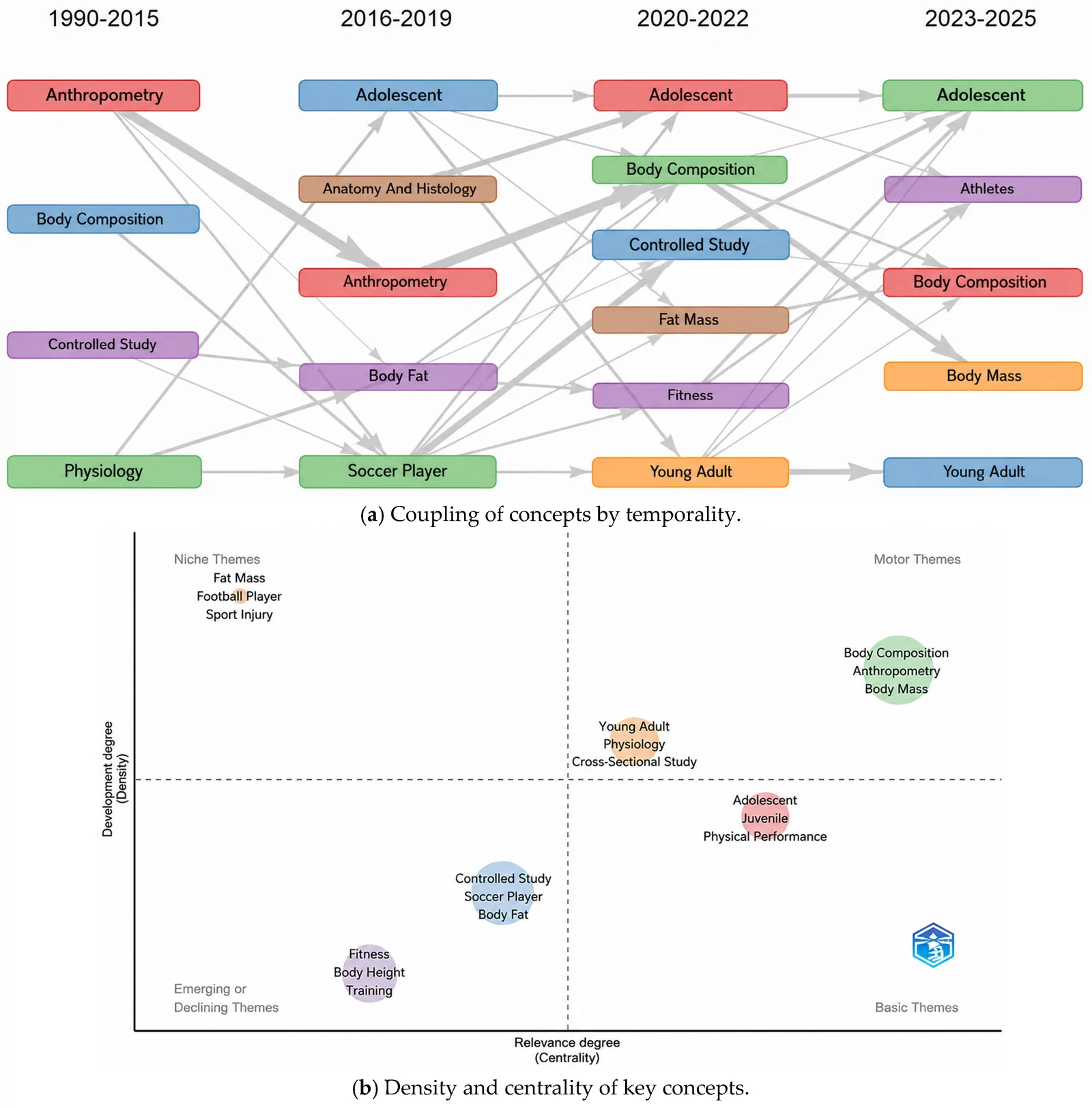
Degree of development and density of key concepts.

Furthermore, Figure 4a reveals the most important concepts used in terms of time. Thus, the coupling clusters show that for the years 2020–2022, the concept of “body composition” had strong associations with “adolescent” and “young adult” in relation to the samples and populations evaluated, while the studies focused on relating anthropometric characteristics to concepts such as “physical fitness,” and the study of fat mass in controlled studies. In general terms, the map shows that research on body composition in the context of sports is structured primarily around anthropometry, body mass, and body composition, which constitute the conceptual core of the field. At the same time, there is a well-established line of research on soccer players, body fat, and sports injuries, albeit with a highly specialized focus. Furthermore, the literature focuses primarily on adolescent populations, wherein physical performance is a recurring variable of interest. Finally, variables related to training and height, and some controlled studies appear to be in an early stage of development or to receive less scientific attention (Figure 4b).

**Figure 5:**
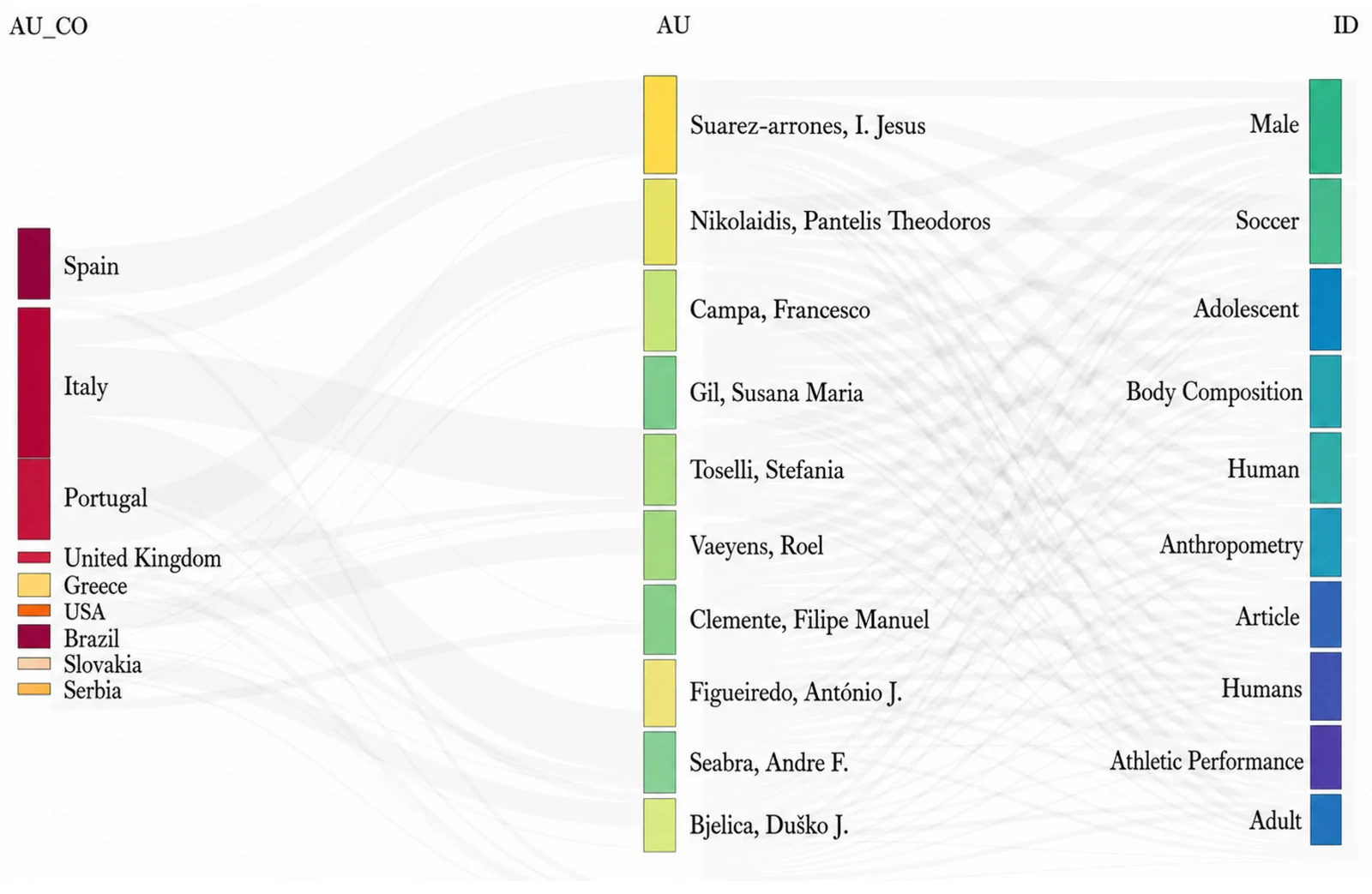
Three-field plot linking author countries, authors, and keywords.

Spain, Italy, Brazil and Portugal stand out as the countries with the highest output. The connections show that some authors collaborate with different countries or engage with various thematic areas, highlighting international research networks. Authors such as Clemente, Figueiredo, Campa, Nikolaidis, and Suarez-Arrones appear to be closely linked to several thematic descriptors, reflecting a broad and diverse body of scientific work. From a bibliometric perspective, the figure shows that scientific research on body composition and anthropometry in soccer players is led by European institutions, particularly those in Italy, Portugal, and Spain. The most productive researchers maintain close ties to the field’s main lines of research, which focus on the assessment of body composition, anthropometry, and physical performance, primarily in adolescent and adult male soccer players (Figure 5). This distribution demonstrates the consolidation of European research groups that have contributed significantly to the conceptual and methodological development of this area of knowledge.

**Figure 6:**
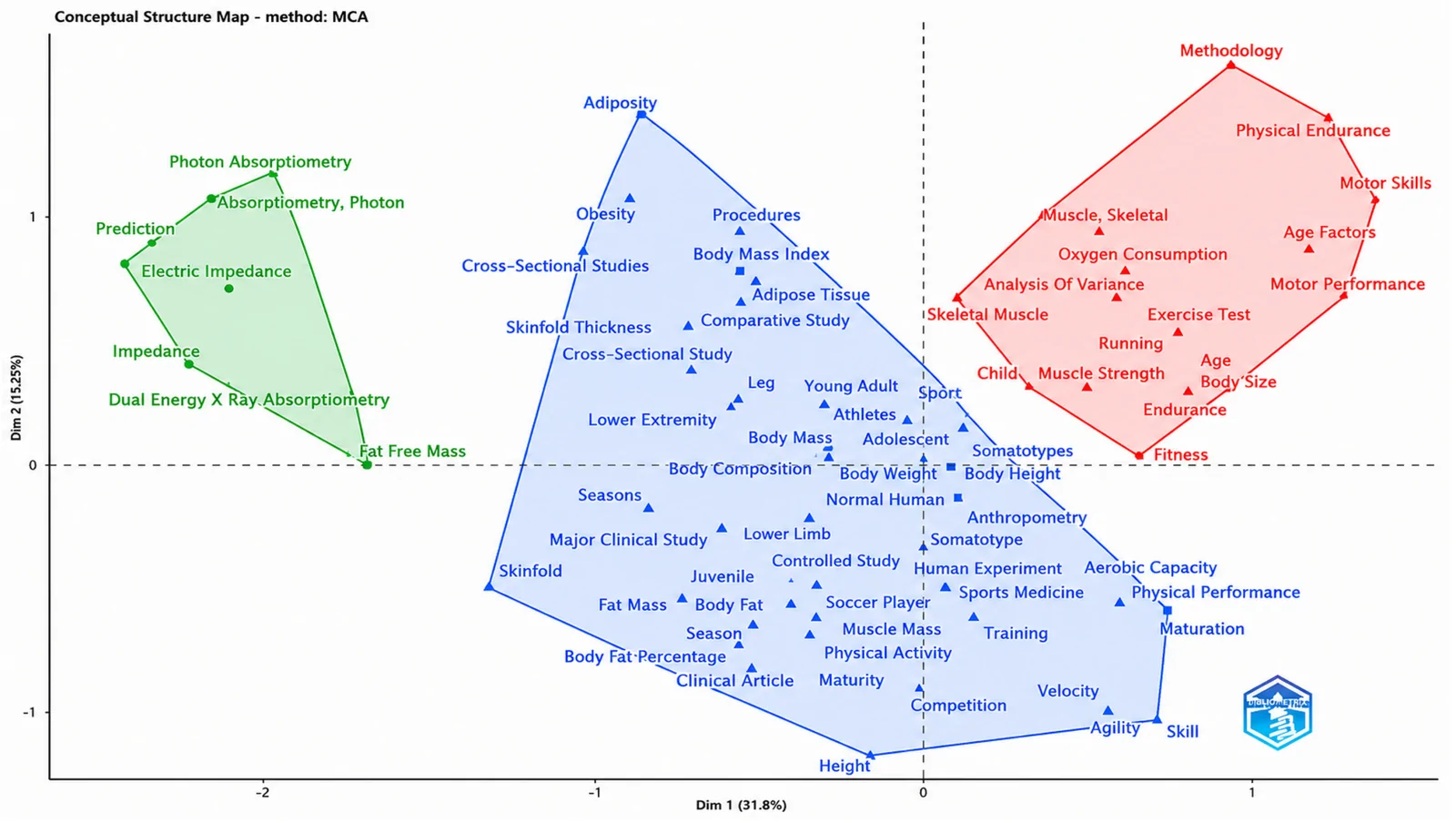
Multiple Correspondence Analysis and dimensionality of key concepts.

As shown in Figure 6, three main thematic clusters were identified. The green cluster groups terms related to body composition assessment techniques, highlighting methods such as photon absorptiometry, DXA, and electrical impedance analysis, reflecting research focused on the validation and comparison of tools for quantifying body composition and fat-free mass. The blue cluster, the largest one, represents the core thematic focus of the scientific output, incorporating concepts such as body composition, anthropometry, body mass index, adolescents, young adults, soccer players, physical performance, and sports medicine. This indicates that most of the literature is oriented toward anthropometric characterization, body composition, and physical performance in athletic populations, particularly young people. Finally, the red cluster brings together terms related to functional assessment and physical performance, including physical endurance, motor skills, motor performance, muscle strength, fitness, exercise test, and oxygen consumption, indicating a line of research focused on physical abilities and their relationship to body composition.

Overall, the conceptual framework shows that research in this field is organized around three complementary areas: (i) methods of body assessment, (ii) anthropometric characterization and body composition in athletes, and (iii) assessment of physical performance and functional abilities. Likewise, Multiple Correspondence Analysis reveals the trend in the concepts used (Figure 6). Dimensions 1 and 2 account for 31.8% and 15.25% of the total information. Therefore, they account for 47.05% of the total inertia. Three main categories were identified: the turquoise category, which associates somatotype with physical fitness capabilities, such as aerobic capacity, agility, and speed, based on cross-sectional studies and a major clinical study; the red category, which associates factors related to player performance, such as oxygen consumption, motor skills, and muscle strength; and the green category, which integrates specific techniques for body composition assessment, including bioelectrical impedance analysis, photon absorptiometry and DXA.

### 3.4. Author Analysis

**Figure 7:**
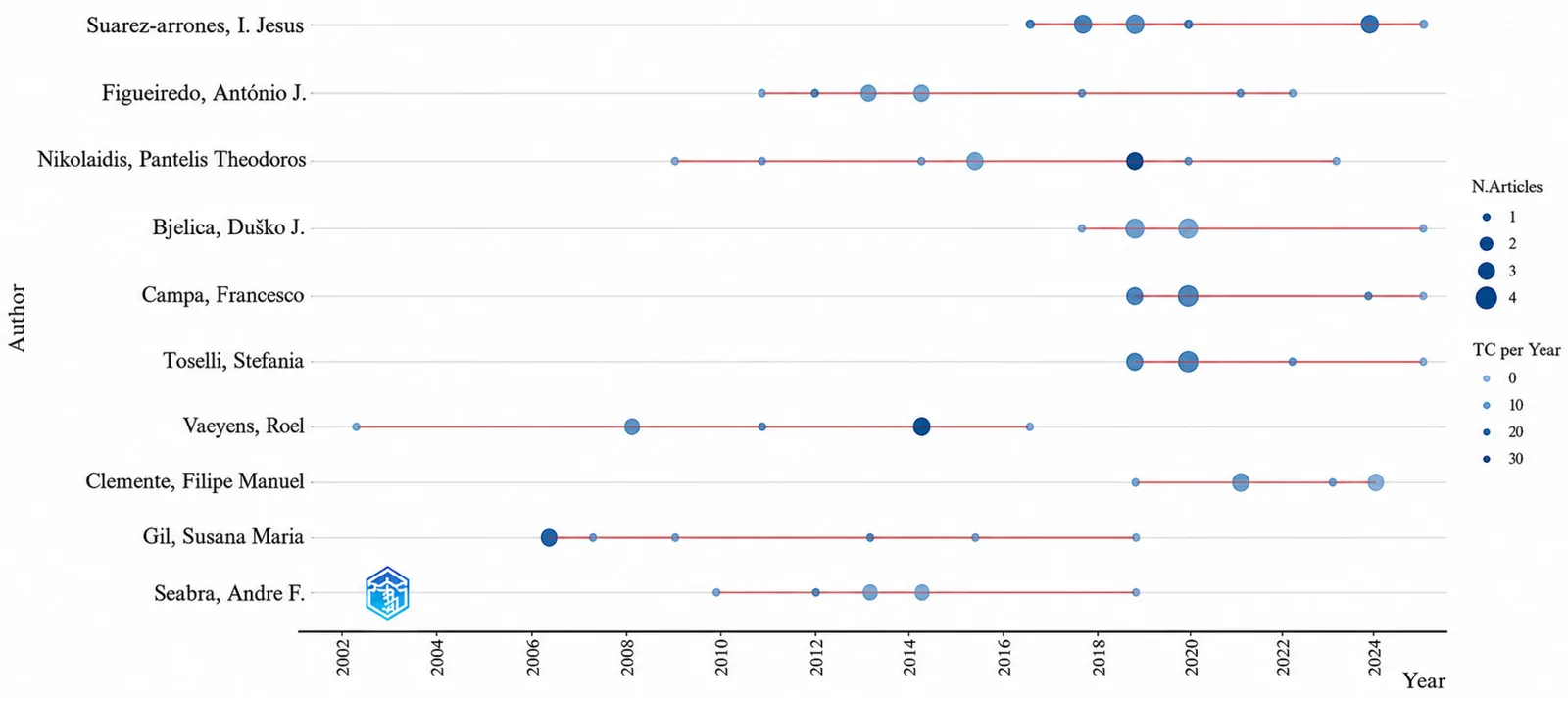
Author production over time (2002–2025) for the most productive and most cited authors.

Figure 7 shows the trend in the scientific output of the authors with the highest number of research papers and citations between 2002 and 2025. Scientific output has clearly increased since 2017–2018, indicating a surge in interest in this topic. It can be observed that the authors Suarez-Arrones, L; Campa, F; and Toselli, S, have led scientific output since 2017. Pantelis Theodoros Nikolaidis, Francisco Campa, Stefania Toselli, and Duško J. Bjelica form a second group of authors who have been highly productive since the late 2010s. Roel Vaeyens, Susana María Gil, and André F. Seabra are pioneering authors whose early publications have had a significant impact, as reflected in their higher citation rates. Overall, the figure illustrates the transition from a field initially developed by a small number of researchers to a broader and more active scientific community, a characteristic of research areas in the process of consolidation.

### 3.5. Keyword Analysis

**Figure 8:**
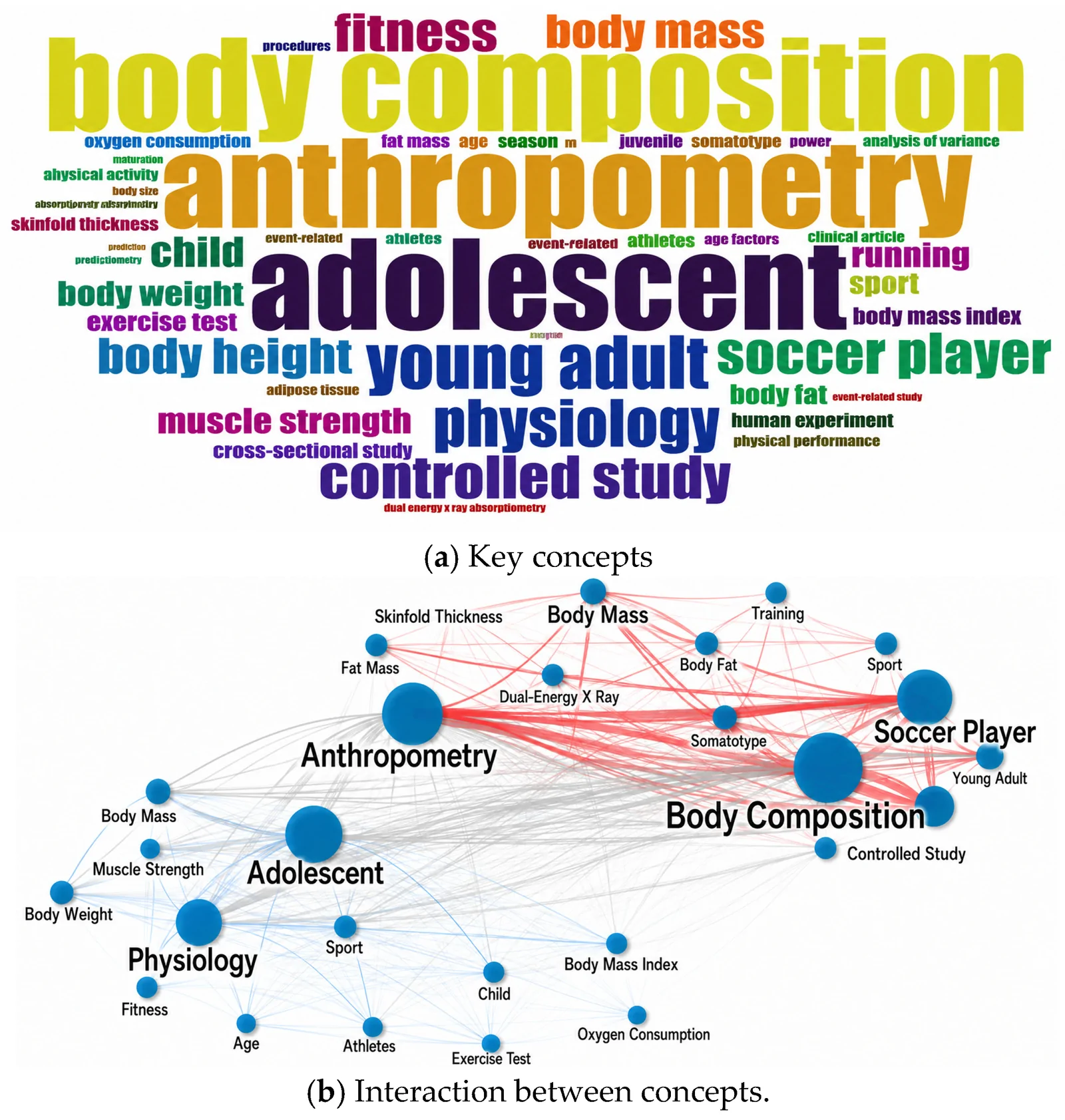
Frequency of occurrence by keywords.

Figure 8a,b shows a keyword co-occurrence network obtained through bibliometric analysis. In this type of visualization, each node represents a keyword; the size of the node is proportional to its frequency of occurrence in the scientific literature, and the lines indicate the strength of the association between the terms. In other words, they reflect the frequency with which the terms appear together in the same documents. The colors of the connections help identify thematic communities or subnetworks within the research domain. Figure 8a shows the keyword map in relation to the most representative concepts and how they relate to each other. In Figure 8a, we observe a wide variety of terms. However, the most frequently used terms are those related to the population: “adolescent” and “young adult”. The high density of connections between body composition, anthropometry, and soccer players indicates that much of the scientific literature focuses on the assessment of the body composition and anthropometric characteristics of soccer players, especially during adolescence and young adulthood. Grouped around these main nodes are terms related to specific body composition indicators (body mass, body fat, body mass index, skinfold thickness, and fat mass), physiological variables (physiology, fitness, exercise test, muscle strength, and sport), and population characteristics (young adult, child, athletes, and somatotype). The close interconnection between these concepts reflects the fact that research takes an integrated approach to analyzing body composition, anthropometry, and physical performance (Figure 8b).

**Figure 9:**
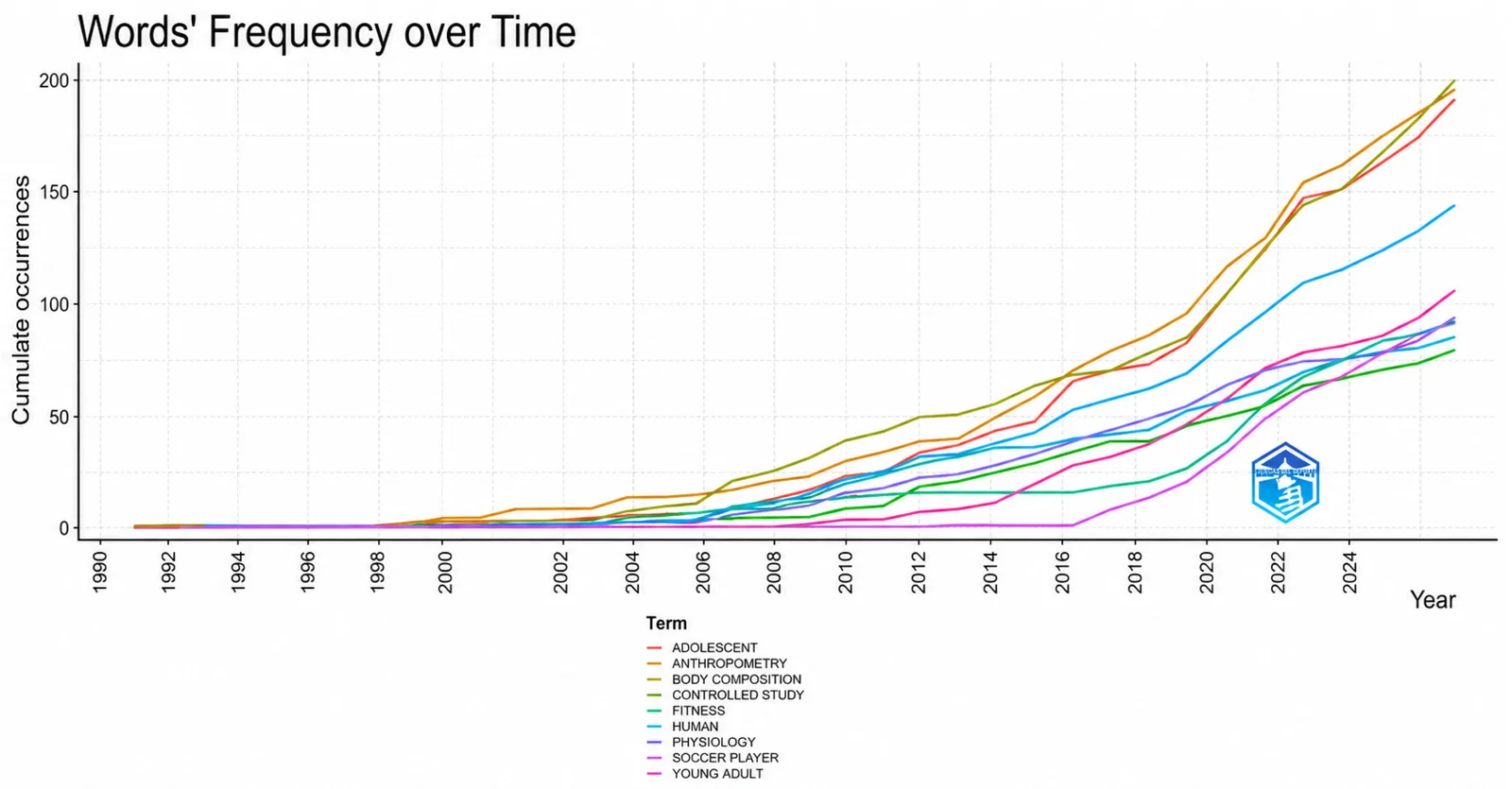
Evolution of key concepts between 1990 and 2025.

Figure 9 shows the frequency of the most frequently used words in scientific publications over the years. The ten words have experienced exponential growth since 2020. The ones that stand out the most are “adolescent,” “anthropometry,” and “body composition,” with a cumulative frequency of nearly 200. The term “soccer player” is noteworthy because it is the only one that only began to take off in 2015, but by 2025, it reached frequencies like those of the other terms.

### 3.6. Analysis of Journals

**Figure 10:**
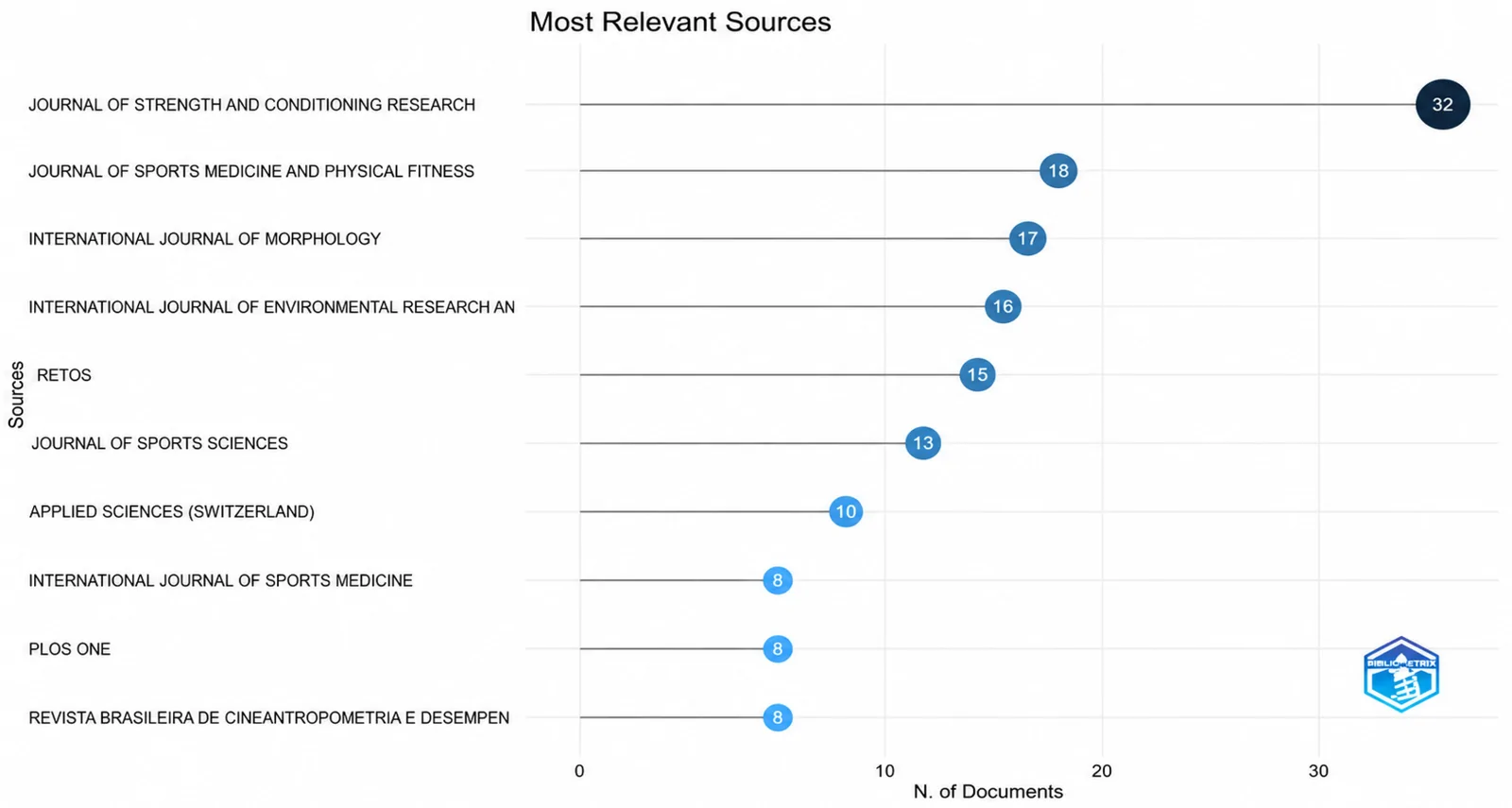
The journals with the highest number of publications in the field of body composition and anthropometry in soccer.

Figure 10 presents the 10 most influential journals based on the number of publications over time, highlighting their output in the field of body composition and anthropometry in soccer. It should be noted that, among these 10 leading journals, only one publishes studies in Spanish, and there is only one publishing in Portuguese.

### 3.7. Country Analysis

**Figure 11:**
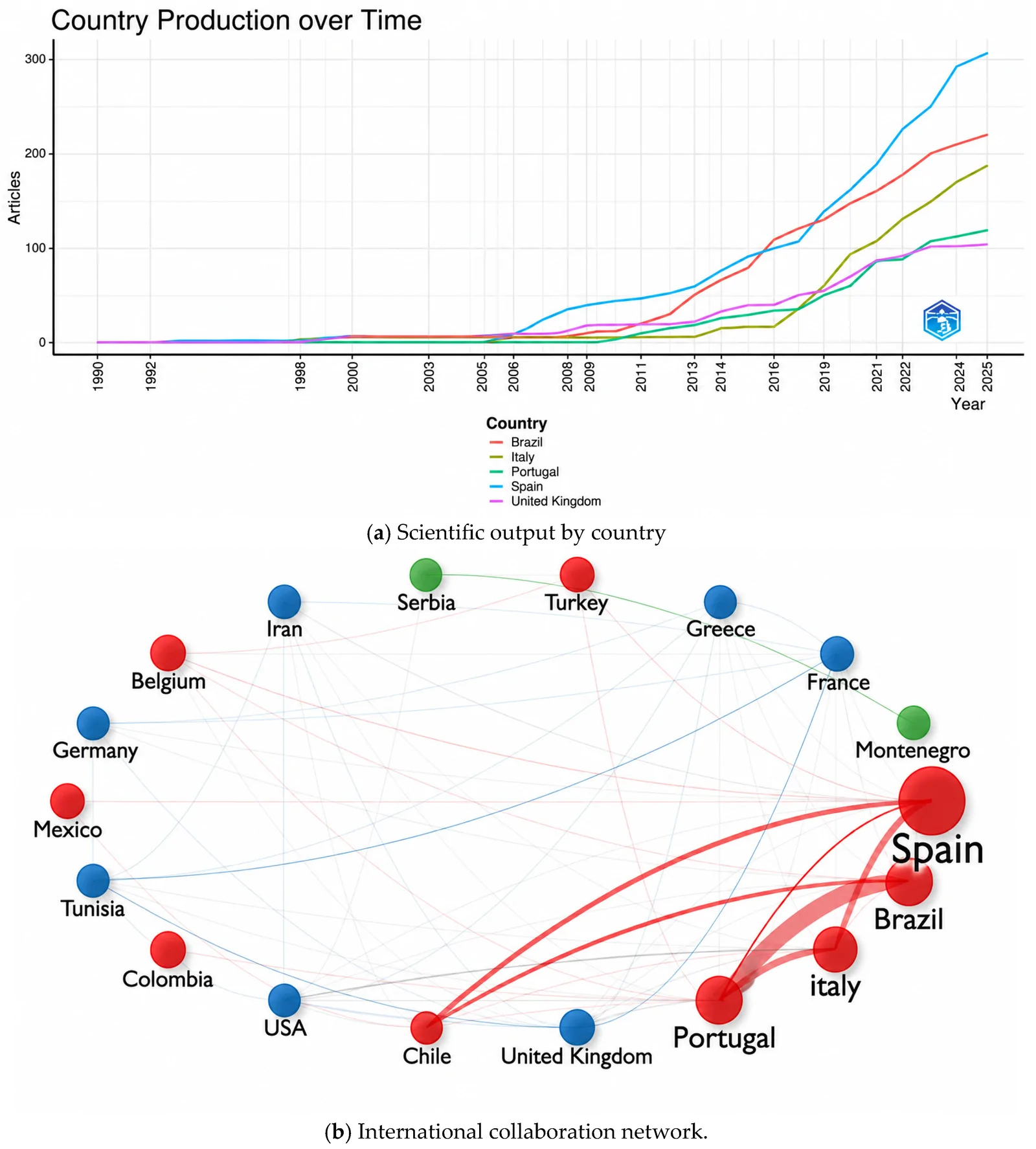
Production and citations by country according to density.

Figure 11a shows the four most productive countries: Spain, Italy, Brazil and Portugal, which represent two continents: Europe and South America. Spain and Brazil have led scientific output since 2013. However, countries like Italy have increased their scientific output in this field since 2019. This reveals the importance that the study of body composition and anthropometry in soccer holds for certain countries. Likewise, Figure 11b shows the interactions between different countries in defining international academic cooperation. The connections established between countries such as Spain, Italy, Brazil and Portugal are highlighted, as they are the ones that generate the highest output and the greatest number of connections.

### 3.8. Organizations

**Figure 12:**
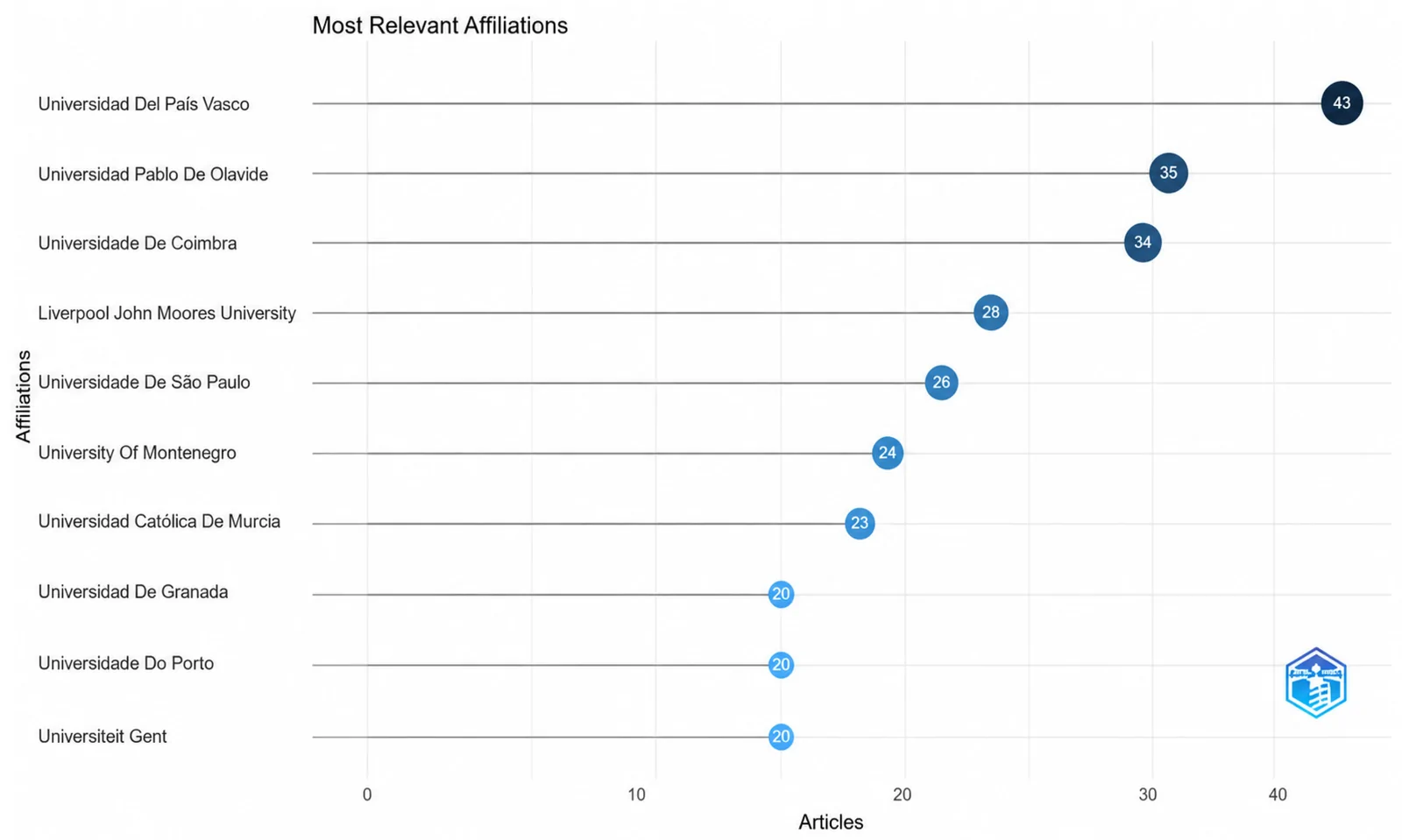
The most important affiliations in the fields of body composition and soccer.

Regarding institutional affiliation, the University of the Basque Country, Pablo de Olavide University, and the University of Coimbra have contributed the most. From 2019 onwards, a high level of institutional affiliation can be observed for countries such as Spain, Portugal, and Brazil (Figure 12). This affiliation chart allows for the visual identification of the institutions with the highest scientific productivity and recognition in the field.

### 3.9. Protocols

Table 2 shows the percentage distribution of the methods and protocols used in the selected studies (n = 384). According to this table, there is significant heterogeneity in body composition assessment procedures. Notably, most studies, despite using validated instruments, do not mention a standardized protocol for performing the assessments (32.29%). Failure to report the protocol used makes it difficult to replicate a study and leads to methodological gaps for future research, such as systematic reviews; due to methodological quality issues, this type of article may present challenges regarding the quality of anthropometric measurements. Furthermore, the most frequently used instruments are bioelectrical impedance analysis (12.76%) and DXA (8.07%). Among the documents describing standardized procedures, the ISAK methodology is the most common, but it does not specify the level of certification for professionals (15.88%). These findings indicate that most studies lack a clear overview of the protocols used to measure body composition.

Furthermore, combined protocols using methodologies such as ISAK, bioelectrical impedance, and DXA were observed; however, the results indicate that none exceeded 1%. Another finding is that ISAK accreditation by level showed varying frequencies, with most studies being level 2 studies (6.51%), followed by level 3 (2.34%) and, finally, level 1 (1.82%). Despite mentioning evaluators’ certification levels, these studies do not specify whether a restricted or full profile was used or whether other specifications established by ISAK through its IsakMetry platform were employed. Traditional standardized protocols such as the International Biological Program (2.34%) and the Anthropometric Standardization Reference Manual (1.30%) were also reported. Therefore, the information supports the need to improve methodological reporting quality in future research.

### 3.10. Population and Sample

Table 3 presents the distribution of the populations in the 384 selected documents. It can be observed that most of the literature focuses on youth players (29.16%), followed by elite players (22.91%) and professional players (14.84%), where the focus is on those aged 14 to 19 years and at highly competitive levels, seeking peak performance. However, the literature also reports on college players (7.29%) and children (5.46%). These results show significant interest in understanding body composition in soccer at both the youth and elite levels.

Research on Amateur players accounted for 3.12%, and sub-elite players for 2.60%. Few studies have focused on comparing outcomes across categories within the same study, such as comparing children to elite players or children to professionals. From this, it can be concluded that soccer has been reported across a wide range of populations but that it is concentrated on the formative and performance stages.

## 4. Discussion

This study offers the first comprehensive bibliometric analysis of body composition and anthropometry in soccer, covering a 35-year period (1990–2025) through a review of 10,300 documents extracted from five international databases. The 384 included studies allow for a rigorous characterization of the productive, thematic, and collaborative dynamics of this field of knowledge. The results of the article are discussed below, section by section.

### 4.1. Scientific Production and Document Typology

The systematic increase in scientific output observed since 2016 is consistent with the global expansion of sports science research over the last decade [24]. This phenomenon coincides with the consolidation of accessible tools for anthropometric assessment in sports contexts as well as with the increased demand for empirical evidence to inform decision-making in professional soccer [17,19]. The predominance of articles (96.09%; n = 369; 9083 citations) over reviews, book chapters, and conference proceedings reflects a scientific community focused on generating primary data, which is to be expected in a field where the diversity of populations, methods, and competitive levels requires specific and contextualized evidence [46,47].

The citation pattern, with a peak in 2000 and relatively low levels since 2015, can be partly attributed to the citation-aging effect: older articles accumulate more citations due to their greater age, while more recent ones are still in the accumulation phase. This does not imply a reduction in the field’s relevance; rather, it is a structural feature of bibliometric growth [48,49,50].

### 4.2. Emerging Issues and Thematic Analysis

The analysis of thematic trends reveals that the concepts of body composition and anthropometry constitute the stable semantic core of the field, while emerging themes include diet, performance among young athletes, and the use of cross-sectional studies. This suggests a progressive specialization in the study of body composition as a longitudinal monitoring variable and as an indicator of response to nutritional interventions [22]. The identification of fat mass and sports injuries as emerging specialized themes is particularly relevant, as it establishes a link between body composition and injury risk, a high-impact line of research applied to high-performance sport.

Multiple Correspondence Analysis confirmed the existence of at least three main dimensions in the literature: one focused on somatotype and physical fitness (aerobic capacity, agility, and speed), another centered on neuromuscular performance (oxygen consumption, muscle strength, and motor skills), and a third focused on body composition assessment techniques (bioimpedance, DXA, and absorptiometry) [51]. This three-dimensional division reflects the field’s maturity and its multidisciplinary integration.

### 4.3. Featured Authors, Countries, and Institutions

The most prolific authors, Suarez-Arrones [52,53,54], Campa [55,56,57], and Toselli [58,59,60], have concentrated their output since 2017 and represent European research groups with a strong tradition in kinanthropometry and exercise physiology. The dominance of Spain, Italy, Brazil and Portugal as leading countries coincides with their soccer and scientific traditions as well as the availability of clubs and academies that facilitate access to high-level competitive samples [9,31]. At the institutional level, the University of the Basque Country, Pablo de Olavide University, and the University of Coimbra emerge as the most active nodes in the co-authorship network, suggesting there are established groups with specific lines of research in this area.

International collaboration networks reveal strong connections between Spain, Italy, Brazil and Portugal, indicating the existence of informal consortia that promote multicenter production. The inclusion of Chile is noteworthy, given that other Latin American countries, aside from Brazil, are beginning to gain visibility in sports anthropometry research [16,32].

### 4.4. Keywords and Semantic Analysis

The prevalence of demographic terms such as adolescent and young adult as the most frequent keywords is consistent with the finding that youth players constitute the main study population (29.16%). This reflects the scientific interest in characterizing morphofunctional development during formative stages, a time when body composition is particularly dynamic and sensitive to the training stimuli associated with biological maturation [1,20]. The association between body composition and physiology, evidenced in keyword co-occurrence maps, suggests that anthropometry is rarely studied in isolation but rather integrated with physiological and performance variables.

### 4.5. Evaluation Protocols and Methodological Heterogeneity

One of the most relevant findings of this study is the high degree of methodological heterogeneity in body composition assessment protocols. The fact that 32.29% of the studies did not specify any standardized protocol represents a critical limitation for the comparability of results between studies. Furthermore, this compromises their reliability due to failure to report the Technical Error of Measurement (TEM), a fundamental parameter for validating evaluator precision. This situation was previously reported by Ackland et al. [17], who emphasized the need to reach a consensus on methodological standards for body composition assessment in sports. The data from this study suggest that this problem persists more than a decade later.

The ISAK-related protocols appear in 29.95% of all included studies and show an unbalanced distribution across certification levels, with level 2 being more frequent (6.51%) than level 1 (1.82%) and level 3 (2.34%). In contrast, bioelectrical impedance (12.76%) and DXA (8.07%) are the most frequently used instrumental methods, possibly due to their accessibility, reproducibility, and ease of application in field settings [4,5]. The low prevalence of combined methods, with no combination exceeding 1%, limits the possibility of cross-validation between techniques.

In close connection with the lack of standardization of protocols, the control and reporting of the TEM as an index of precision and data quality become of vital importance [61]. Anthropometry is a technique intrinsically susceptible to measurement errors derived from observer variability, the use of equipment, methodological inconsistencies, and, above all, imprecision in the correct localization of anatomical sites [62,63]. A lack of precision in these measures directly affects the reliability and accuracy of body composition estimates, which can weaken or distort the associations observed in applied research [63]. For this reason, strict adherence to normative protocols (such as those of the ISAK) and the continuous training of anthropometrists are fundamental to quantify and minimize the variability in the TEM in both an intra- and inter-evaluator manner [62]. Maintaining the TEM within scientifically acceptable levels or thresholds of tolerance guarantees that the extracted profiles are highly reproducible, thereby ensuring that the differences detected in the subjects correspond to a real biological or morphological variation and not a systematic or random error introduced by the evaluator [61].

Beyond the reported differences, a methodological problem or limitation emerges in relation to how the concept of nutritional status is operationalized in the literature [64]. Many studies rely on indirect or two-compartment methods, such as bioelectrical impedance analysis (BIA) and DXA, which, although practical, have limitations, including their sensitivity to hydration status and assumptions about constant tissue densities [65], which compromise their validity for athletic populations [17]. This is insufficient for elite athletes, whose body composition deviates from general population models [66].

Thus, the limited adoption of multicompartmental partitioning approaches such as the Kerr model suggests a gap between methodological advances in kinanthropometry and their implementation in applied research [61]. These models allow for a more precise measurement of body mass in structural components (e.g., adipose tissue, muscle, bone, residual, and skin) and promote reporting in both absolute (kg) and relative (%) terms, improving interpretability in high-performance contexts [17,67].

### 4.6. Competitive Levels of Populations

The concentration of studies on youth (29.16%) and elite (22.91%) players confirms that scientific interest is focused on the stages of the greatest morphofunctional plasticity and highest competitive impact. However, the underrepresentation of female players identified in the literature, serving as a structural problem in the field [28,29], constitutes a bias that future research should address. Furthermore, the lack of comparative studies across multiple competitive levels limits our understanding of body composition development trajectories throughout an athletic career.

### 4.7. Limitations

This study has some limitations that should be considered when interpreting its results. First, the search was conducted across five reference databases, which, while ensuring broad coverage, may have excluded studies published in regional repositories or in languages other than English, Spanish, or Portuguese. Second, the bibliometric analysis is based on the metadata available in the databases consulted, so errors or inconsistencies in keyword assignment, institutional affiliations, or citation data could affect specific results.

Third, while the Bibliometrix R tool is the standard in the bibliometric literature, it has limitations in processing documents with incomplete information, which could have affected the cleaning of the initial 10,300 documents. Fourth, the analysis of the evaluation protocols was based on information reported within the studies themselves, but we could not access the full texts of all the included documents, so we could have underestimated the true prevalence of certain methods. Fifth, the cross-sectional and descriptive nature of bibliometric analysis does not allow for the establishment of causal relationships or for drawing conclusions about the methodological quality of the reviewed studies relative to other types of assessments to methodologically determine which is more effective for analyzing body composition. Finally, other bibliometric studies have highlighted the limitations of the databases, which can directly affect the stored results, as these also depend on the homogenization of the data, extraction, and analysis software.

### 4.8. Practical Applications

The results of this study provide practical information for the various stakeholders in the scientific and sports ecosystem. For researchers, keyword co-occurrence maps and emerging-theme analysis allow identification of underdeveloped lines of research, such as body composition of referees, futsal, or comparisons between sports modalities, which represent concrete opportunities to generate original contributions to the field. This is primarily due to the gap in the corpus resulting from the design used.

For sports professionals, including strength and conditioning coaches, nutritionists, kinesiologists, and sports physicians, the findings on protocol heterogeneity reinforce the need to adopt standardized methodologies, such as the ISAK guidelines, in their routine assessments. Furthermore, it is imperative to report the TEM and specify the type of profile applied to determine the anthropometric characteristics of the target population, moving beyond simply reporting the anthropometrist’s accreditation level. This facilitates comparability with international literature and knowledge transfer between clubs and institutions. Our identification of BIA and DXA as the most widely used methods can guide investment decisions in body composition assessment equipment in high-performance settings.

For editors and reviewers of scientific journals, the data show that 32.29% of studies lack specified protocols the importance of including explicit criteria in methodological reports, both in author guidelines and in peer review processes. It would be advisable to request a declaration of the assessment protocol used, the assessor’s certification level, and the instrument employed as minimum requirements for methodological quality.

Finally, for soccer governing bodies, Fédération Internationale de Football Association (FIFA), Union of European Football Associations (UEFA), and national federations, the geographical distribution of scientific output demonstrates the need to promote research in underrepresented regions, such as Africa, Asia, and Central America, to ensure that knowledge about body composition in soccer reflects the ethnic, cultural, and socioeconomic diversity in the global practice of this sport.

## 5. Conclusions

The present bibliometric analysis of body composition and anthropometry in soccer (1990–2025) allows us to draw the following main conclusions from the 384 included studies. Although the first paper in this field was published in 1990, scientific output has experienced growth since 2016, with 14 papers published that year, establishing it as a highly active area of research within the sports sciences. The included studies, mostly articles, have accumulated over 9400 citations, demonstrating the topic’s impact and relevance within the international scientific community.

Spain, Italy, Brazil, and Portugal lead global production in collaboration and partnerships with institutions such as the University of the Basque Country, Pablo de Olavide University, and the University of Coimbra, which serve as the most active hubs. Strong international collaboration networks exist between European and Latin American countries, although representation from regions such as Africa and Asia remains limited, presenting an opportunity for future development.

Emerging topics, such as diet, youth players, fat mass, and sports injuries, point to an evolution toward a comprehensive conception of body composition, articulated with nutrition, injury prevention, and training periodization, opening opportunities for longitudinal and multidisciplinary research.

Methodological heterogeneity in assessment protocols constitutes the main structural weakness of the field: 32.29% of studies do not specify their protocols, compromising the comparability of results. The widespread adoption of international standards, such as the ISAK methodology, along with mandatory disclosure of the instrument used and the assessor’s certification level, is presented as a priority for improving the quality of scientific reporting in future research.

## Figures and Tables

**Table 1:** Types of documents published.

Document Type	Number of Documents	Percentage (%)	Number of Citations	Percentage (%)
Article	369	96.09	9083	96.19
Review	5	1.30	325	3.44
Chapter of a book	4	1.04	1	0.01
Conference presentation	3	0.78	27	0.29
Letter	3	0.78	6	0.06
Total	384	100	9442	100

**Table 2:** Protocols and instruments for body composition analysis.

Protocol	N	%
It is not specified	124	32.29%
The ISAK protocol is not specified	61	15.88%
Bioelectrical impedance	49	12.76%
No access	37	9.64%
DXA	31	8.07%
ISAK 2	25	6.51%
ISAK 3	9	2.34%
International Biological Program	9	2.34%
ISAK 1	7	1.82%
Anthropometric Standardization Reference Manual	5	1.30%
Air displacement plethysmography	4	1.04%
ACSM	2	0.52%
During and Webster	2	0.52%
ISAK 1 for bioelectrical impedance	2	0.52%
ISAK protocol that is not specified and DXA	2	0.52%
ISAK protocol specific to bioelectrical impedance	2	0.52%
DK-ANT	1	0.26%
DXA and direct calorimetry	1	0.26%
Bioelectrical impedance and DXA	1	0.26%
Bioelectrical impedance and the International Biological Program	1	0.26%
ISAK 1 and 3	1	0.26%
ISAK 1, DXA, and bioelectrical impedance	1	0.26%
ISAK 2 and 3	1	0.26%
ISAK specific to air displacement plethysmography	1	0.26%
ISAK protocol is specific; DXA is for bioelectrical impedance	1	0.26%
ISAK and EGK	1	0.26%
ISAK, bioelectrical impedance, and DXA	1	0.26%
Air displacement plethysmography and DXA	1	0.26%
SATA III and IV	1	0.26%
Total	384	100%

Note: ISAK: International Society for the Advancement of Kinanthropometry; DXA: dual-energy X-Ray absorptiometry; ACSM: American College of Sports Medicine; DK-ANT: Droghetti and Knight—Anthropometry; EGK: Spanish Group of Kinanthropometry; SATA: Sistema de Acreditación en Técnicas Antropométricas.

**Table 3:** Distribution and level of competitiveness of the populations.

Population	n	%
Youth players	112	29.16%
Elite players	88	22.91%
Professional players	57	14.84%
College players	28	7.29%
Child players	21	5.46%
Older players	18	4.69%
Amateur players	12	3.12%
Sub-elite players	10	2.60%
Child and youth players	8	2.08%
Semi-professional players	8	2.08%
Youth and veteran players	6	1.56%
Elite and sub-elite players	5	1.30%
Players ranging from children to adults	4	1.04%
Teenage players	3	0.78%
Players ranging from children to professionals	2	0.52%
Players at an unspecified level	2	0.52%
Total	384	100%

## Data Availability

The data presented in this document can be verified based on the results obtained, which can be consulted by contacting the corresponding authors.
